# What is driving the global decline of human fertility? Need for a multidisciplinary approach to the underlying mechanisms

**DOI:** 10.3389/frph.2024.1364352

**Published:** 2024-04-25

**Authors:** Robert John Aitken

**Affiliations:** ^1^ Priority Research Centre for Reproductive Science, Discipline of Biological Sciences, School of Environmental and Life Sciences, College of Engineering Science and Environment, University of Newcastle, Callaghan, NSW, Australia; ^2^ Hunter Medical Research Institute, New Lambton Heights, NSW, Australia

**Keywords:** human population, total fertility rate, urbanization, female education, infertility, reproductive toxicants, natural selection, demographic transition

## Abstract

An intense period of human population expansion over the past 250 years is about to cease. Total fertility rates are falling dramatically all over the world such that highly industrialized nations, including China and the tiger economies of SE Asia, will see their populations decline significantly in the coming decades. The socioeconomic, geopolitical and environmental ramifications of this change are considerable and invite a multidisciplinary consideration of the underlying mechanisms. In the short-term, socioeconomic factors, particularly urbanization and delayed childbearing are powerful drivers of reduced fertility. In parallel, lifestyle factors such as obesity and the presence of numerous reproductive toxicants in the environment, including air-borne pollutants, nanoplastics and electromagnetic radiation, are seriously compromising reproductive health. In the longer term, it is hypothesized that the reduction in family size that accompanies the demographic transition will decrease selection pressure on high fertility genes leading to a progressive loss of human fecundity. Paradoxically, the uptake of assisted reproductive technologies at scale, may also contribute to such fecundity loss by encouraging the retention of poor fertility genotypes within the population. Since the decline in fertility rate that accompanies the demographic transition appears to be ubiquitous, the public health implications for our species are potentially devastating.

## 1 Introduction

Changes in the size of the human population have obvious implications for the environmental status of our planet, the economic growth of its constituent nations, as well as the health and wellbeing of the societies we live in. The factors impacting human population dynamics are complex and involve an intricate interplay of social, economic, environmental, and biological forces. Because of this complexity, predicting the rate and direction of human population change has always presented a difficult challenge and has been mired in controversy (1–8). Current UN projections for the future (United Nations 2022), assume that all nations ultimately converge and stabilize their populations at a total fertility rate (TFR; defined as the total number of children born per woman in a reproductive lifetime assuming current age specific fertility rates) just below replacement level, which approximates to 2.1 children per woman. The TFR at which the population is predicted to stabilize has varied with the passage of time and the source of the prediction, but recent projections suggest a sub-replacement value of 1.66–1.75 by 2100 (7–10). The assumption underpinning such models is that with increasing socioeconomic development we shall see a global reduction in TFR as a direct consequence of the demographic transition model (the reduction in family size that accompanies the decrease in infant mortality triggered by increased economic growth and improved public health awareness). Once TFR has declined to below replacement level, this downward pressure is assumed to somehow relent, allowing the population to restabilize at a point where population decline will be reassuringly slow. The wisdom that generates this state of equilibrium is held to involve a range of social and political factors invoked by society and Government, and designed to provide levels of social security, gender equality, market flexibility and financial reward needed to restoke the fires of fertility (11). Whether control over population dynamics is achievable, depends upon our fundamental understanding of the underlying mechanisms and the willingness of societies and Governments to engage these causative factors and actively manage their population numbers. In this article, I review the range of cultural, social, biological, and environmental factors that are known to impact human fertility and consider whether we yet have the tools and knowledge needed to control population density at both national and global levels.

## 2 The shape of world population growth

**Figure 1:**
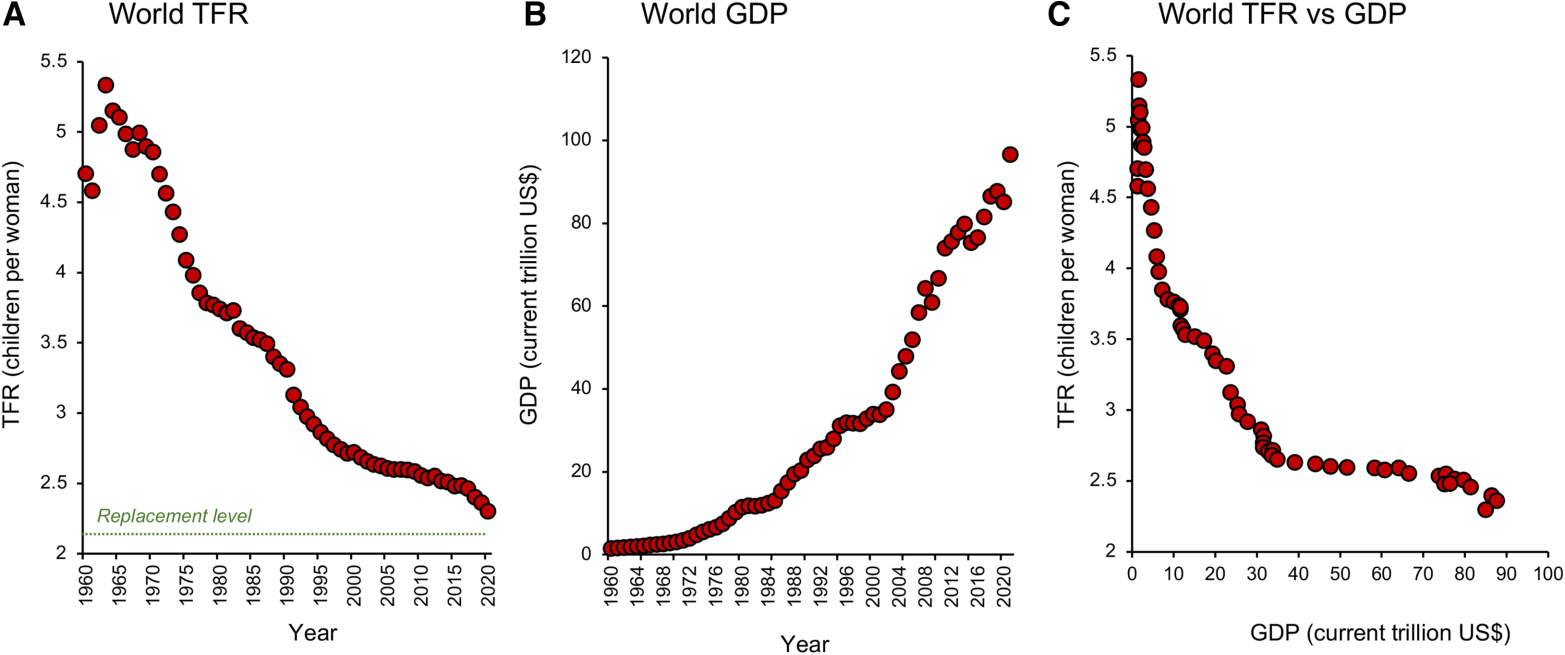
Global changes in total fertility rate and prosperity. (**A**) Since 1960 the world has witnessed a brief surge in TFR (the average number of children a woman is projected to give birth to in her reproductive lifetime) which peaked in 1963 and then commenced a dramatic decline towards replacement level, which is conventionally defined as 2.1 children per woman. (**B**) The fall in TFR was associated with a global increase in prosperity measured in terms of Gross Domestic Product (GDP). (**C**) A plot of TFR against GDP shows that just a modest increase in the latter can precipitate a marked fall in TFR. Source: World Bank Open data retrieved from https://data.worldbank.org/.

It took from the origin of our species to 1 AD to achieve a global population of 150–200 million. Our numbers then grew slowly over time but stalled in 1300 when the black death took its deadly toll across Asia and Europe. Again in 1600, the 30 Years War in Germany and the end of the Ming Dynasty in China caused a momentary reduction in our rate of population growth. In 1850, the rate of global population growth slowed again, possibly because of the Taiping rebellion in China which left up to 70 million dead. Finally, World War 1 and the influenza pandemic that followed accounted for 66 million deaths and briefly slowed the rate of population growth between 1914 and 1919 while World War II accounted for another 70 million souls. These were, however, mere speed bumps on our road to global ascendancy. Once we had learned how to harness the energy bound up in fossil fuels and understood the importance of primary healthcare in controlling infant mortality, there was no stopping us (12, 13). By 1800, the world had reached a population of 1 billion and thereafter rocketed upwards achieving the second billion in only 130 years (1930), the third billion in 30 years (1960), the fourth billion in 15 years (1974), and the fifth billion in only 13 years (1987). A staggering statistic is that in the 1970s, when Paul Ehrlich's book, “The Population Bomb” was opening our eyes to the potential consequences of untrammelled population growth (14), there were roughly half as many people on planet Earth as there are today (3.8 billion in 1971, compared with 7.8 billion today). Clearly Ehrlich's message gained very little political or social traction. The world's population has continued to grow unabated and our attempts to stem this flood with everything from contraceptive vaccines to mass sterilization programs waged by anxious Governments have, in essence, failed. Notwithstanding the failure of such top-down approaches to control fertility, recent data suggest the progressive emergence of a new state-of-affairs. After the post-war period of rapid population growth captured by Ehrlich, the *rates* of population growth started to decline in the 1960s and are predicted to become negative later this century (7). Such a change in population dynamics has been induced by a sudden reduction in fertility rate which has been ongoing for the last half century and is closely correlated with the simultaneous increase in global prosperity (Figures 1A–C) as well as the cultural, social, and economic forces associated with the development of modern society (14–17).

**Figure 2:**
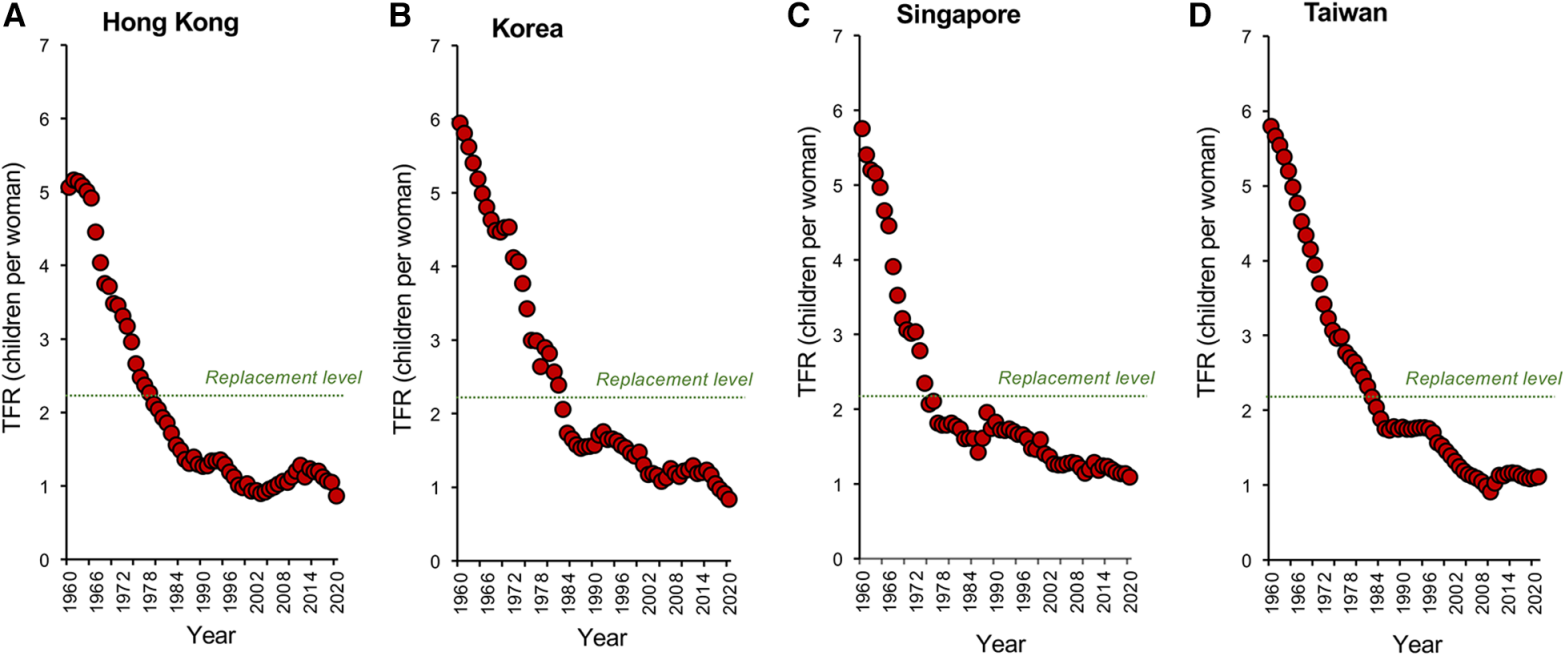
Changes in TFR within the tiger economies of SE Asia. Global fertility decline has been particularly marked in the tiger economies of SE Asia including (**A**) Hong Kong. (**B**) South Korea (**C**) Singapore and (**D**) Taiwan. In all these countries, the dramatic economic growth we have witnessed over the past half century has resulted in a significant decline in fertility rates that have extended to below the replacement level threshold, with no sign of a resurgence. Source: World Bank Open data retrieved from https://data.worldbank.org/.

**Figure 3:**
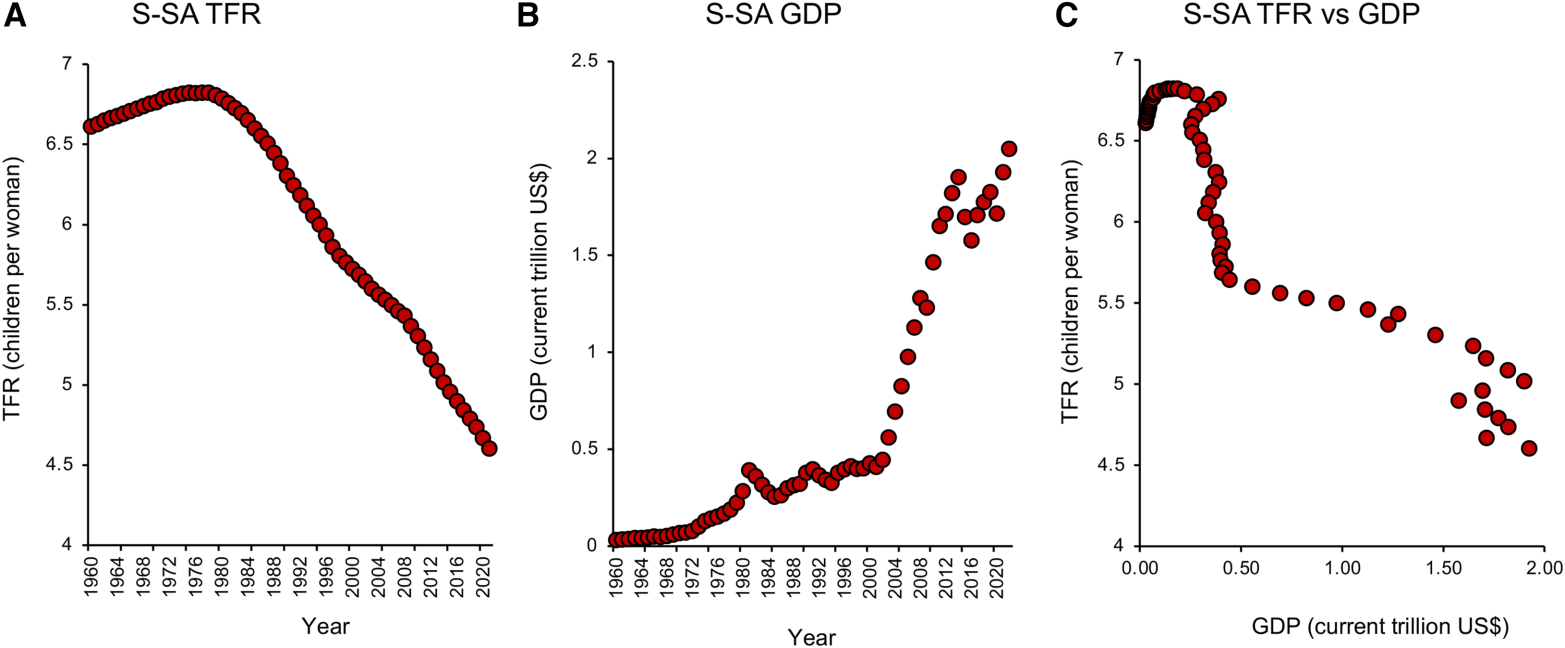
Total fertility rate and GDP in Sub-saharan Africa. (**A**) Changes in TFR from 1960 to 2021 in Sub-Saharan (S-SA) reveal an early rise, followed by a steady fall that shows no signs of abating. (**B**) Over the same period there was a progressive increase in GDP measured in current US$. (**C**) A plot of TFR against GDP indicating that once the limited phase of population growth was over, TFR values fell rapidly in response to a very small increase in GDP. Source: World Bank Open data retrieved from https://data.worldbank.org/.

Nowhere is this decline in TFR more evident than in the tiger economies of SE Asia. Countries like Hong Kong, South Korea, Singapore, and Taiwan (Figures 2A–D) may be affluent, but prosperity has come at a cost to their fertility which, following a period of rapid descent, now ranks amongst the lowest in the world. Not only are the fertility rates in these countries below replacement level, but they are showing no signs of recovery. Moreover, the tiger economies are not alone. Globally, TFRs began to fall in the early 1960s and have ultimately affected every recorded country—from America to Albania, from the United Kingdom to Uzbekistan. According to the United Nations (18), two-thirds of the global population currently live in countries with fertility rates below replacement levels. Even in those areas of the world such as sub-Saharan Africa where fertility rates remain high (19), we see the same general downward trend with the passage of time (20), associated with the modernization of society and a progressive increase in prosperity (Figures 3A–C). Thus, all countries appear to be on the same fundamental demographic journey, it is just that some nations embarked upon their voyage earlier than others—and their ships have different speeds and capacities. The socio-economic, geo-political, environmental, and biological implications of uncontrolled population decline are clearly extremely complex (21, 22). There are clear *advantages* from an environmental perspective just as there are evident *disadvantages* in terms of economic growth and the provision of societal infrastructure. Whether nations want to recover from severe fertility decline (e.g., South Korea, Japan, Singapore) or limit levels of population growth (Sub-Saharan Africa) we need to gain some measure of control over this process. Such initiatives, in turn, requires that we step outside our traditional discipline boundaries and adopt a more multidisciplinary approach to understanding the range of forces that are currently driving the downturn in global fertility. In the following sections, I bring together some of the key social, political, environmental, and biological factors that are shaping human population dynamics and consider their implications for the future.

## 3 Role of governmental policies and contraception

**Figure 4:**
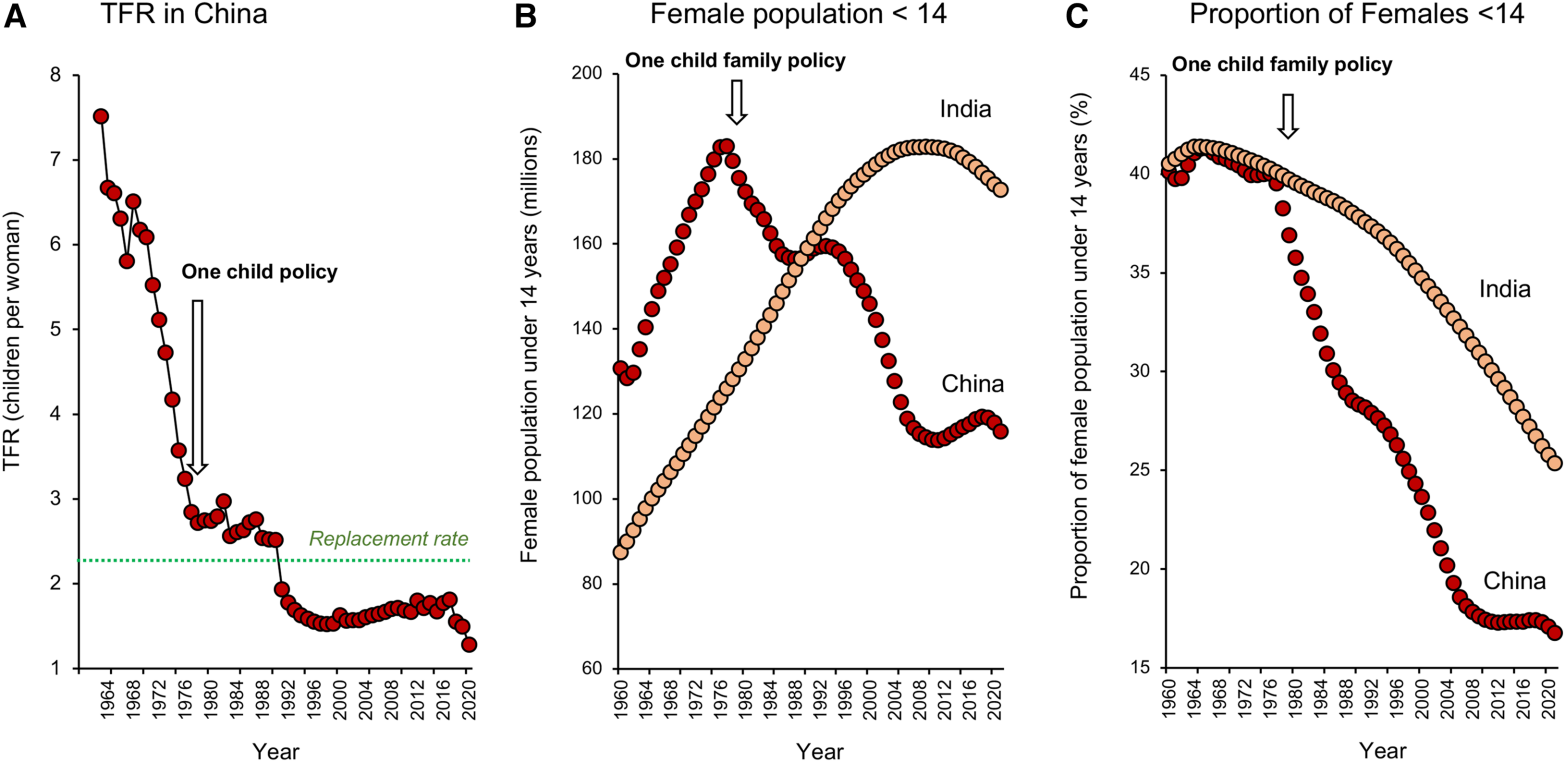
Impact of policy on fertility decline in China and India. (**A**) Fertility rates were falling in China well before the one-child-family policy was introduced in 1979–1980. (**B**) The one-child-family policy did however lead to an absolute decline in the number of young girls less than 14 years of age in the Chinese population, whereas this cohort increased in size in India, peaking around 2011. (**C**) When expressed as a function of the total female population, the proportion of girls less than 14 years old declined in both Indian and Chinese populations because the demographic transition led to a change in age structure favouring the older adult. However, the impact of the one-child-family policy is evidenced by an acceleration in the rate at which the proportion of young females in the overall population decreased in China, seriously compromising this country's demographic momentum. As a result, population numbers will decline more rapidly in China than India. Source: World Bank Open data retrieved from https://data.worldbank.org/.

As we shall see later in this article, Government policies can have a major impact on managing the *consequences* of global fertility decline, but do they pay any role in its *induction*? In terms of political participation in demographic matters, many might consider China as a special case characterized by the one-child-family policy, brought in by a government desperate to rein in unsustainable population growth. However, a consideration of the data (Figure 4A) reveals that fertility rates in China were actually in rapid decline long before the one-child-family policy was introduced in 1979–80. It is possible that the preceding “*later, longer, fewer”* (LLF) campaign (1973–1979), aimed at encouraging later first births, longer birth intervals and fewer children overall, had an impact on TFR (23). However, fertility rates had already started to decline by 1973 and while government policy has clearly played a role, it is the Chinese people who have been the major architects behind this demographic change. As Feng (24) eloquently puts it, “*in terms of both origin and agency, it is the Chinese people not the Chinese state, who are the main motors behind China's demographic transition*”.

Even if the one-child-family policy did not have a major impact on TFR in China, it *was* highly successful in reducing population momentum. The latter defines the means by which populations can continue to grow even though their TFR has fallen to below replacement levels (25). It is a buffering mechanism whereby populations can harvest the benefits of their previous fecundity, as a result of the continuing entry of young women into the reproductive age cohort. Following the introduction of the one child family policy in China, the number of young females less than 14 years of age in the population declined rapidly and now stands at 116 million, representing around 17% of the total female population. By contrast, in India, the absolute number of young females grew steadily until around 2011 (Figure 4B). While population ageing in India has seen the proportion of the female population under 14 years of age decline to around 25%, there are still 173 million girls in this category. This gives the Indian population significant momentum and explains why it will continue to increase in the coming decades to become the world's most populous nation, even though total fertility rates have been declining for some time. In contrast, the Chinese population is already beginning to shrink (26) and the point of deviation between these two populations coincides with the introduction of the one child family policy (Figure 4C).

China apart, any government that introduces policies that increase the cost of having children and/or the proportion of women in the workforce, has the potential to contribute to a decline in TFR (27). The overall state of the economy is inevitably a major contributor to declining TFR as exemplified by the negative impact of economic recessions that look place in Spain in 2008 (28) and between 1998 and 2013 in the poorer areas of Columbia (29). Conversely, there are many examples of pronatalist policies that can have a positive impact on fertility including: baby bonuses in the form of cash transfers and tax incentives (30), Scandinavian-style parental leave schemes that provide parents with the opportunity to care for their child without suffering career disadvantage (31), provision of adequate childcare facilities (32) and the supply of affordable housing (33, 34).

**Figure 5:**
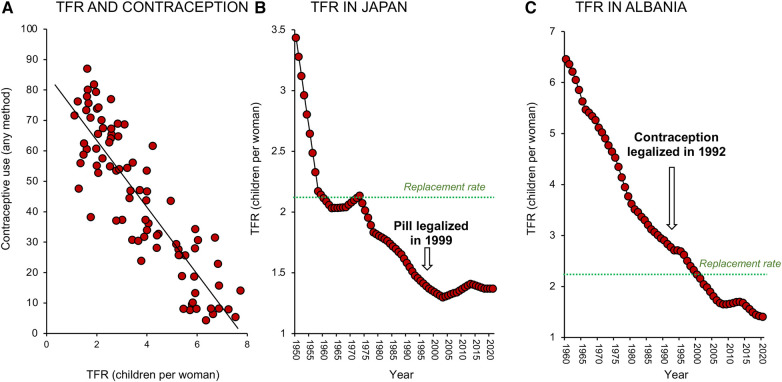
Impact of contraceptive use on fertility decline. (**A**) There is a clear negative correlation between TFR and contraceptive use; data for 2000–2001. (**B**) The relationship between contraceptive use and TFR is not necessarily causative. Fertility rates fell in Japan long before the oral contraceptive pill became available in 1999. (**C**) Similarly, in Albania fertility rates fell dramatically between 1960 and 2020 and were not significantly impacted by the legalization of contraception in 1992. Sources: United Nations Department of Economic and Social Affairs, Population Division (18) and World Bank Open data retrieved from https://data.worldbank.org/.

If there is one area where Government policy that should have a consistent impact on TFR, it is in the provision of effective family planning services (35, 36). The wealthier and more urbanized a population becomes, the more available are the contraceptive means to control reproduction, and fertility rates would be expected to decline as a consequence. Indeed, if we take a snapshot of global fertility rates in 2000–2001, and plot these values against contraceptive use by country, a clear negative correlation is observed (Figure 5A; *R*^2^ = 0.775); the greater the uptake of contraception, the lower the fertility rate.

Such correlations are rational, but is the relationship causative? In Japan for example, the oral contraceptive pill was not legalized until 1999 and yet fertility rates were falling decades before the pill became available (Figure 5B). In other countries like Albania (Figure 5C), fertility rates declined dramatically between 1960 and 1990 even though contraception was not legalized in this country until 1992. Strongly Catholic countries like Mexico, Brazil, Paraguay, and the Philippines all experienced a dramatic decline in TFR in the 1960's and 70s, despite the lack of any official support for “unnatural” methods of family planning (37). Since, as a species, we are not much given to abstinence, it is safe to assume that contraception is inevitably involved in the global decline in TFR (38). However, it is not necessarily the primary driving force; in many of the world's most advanced societies contraception is simply an enabling mechanism that supports the societal need for fertility change. In modern affluent societies where women have the freedom to make autonomous choices about their reproductive health, contraceptive use constitutes a means of temporarily suppressing fertility in favour of education and professional career development (39). In some situations, contraceptive uptake may also be coercively induced as a matter of governmental policy or in response to numerous social, cultural, and institutional factors (40, 41). However in general, the increasing availability of contraceptives is not the primary driver for fertility decline. It is an important component of the fertility equation and a major enabler of low fertility rates in advanced economies. However, it is a correlate rather than a cause of the global TFR change depicted in Figure 1.

## 4 Food availability

In 1798 the English Cleric, Thomas Malthus published his seminal work “*An Essay on the Principle of Population”* describing the complex relationship between resource availability and population growth; he was just 32 years old at the time (42). In this essay he pointed out that as nations prospered, and food supply became abundant, the population naturally tended to grow. However, while population expansion tended to follow an exponential, geometric progression, the resources needed to support the population only increased in a linear, arithmetic fashion. As a consequence, when human populations expand, the per capita resource base shrinks and the lower classes suffer hardship as a result of disease and starvation. Malthus concluded “*I see no way by which man can escape from the weight of this law which pervades all animated nature*”. This argument was appropriated by Paul Ehrlich in the 1960s who essentially posited that population growth was so rampant that it would eventually outstrip our capacity for food production and so, ultimately, hunger would define our destiny. Like many of the assertive statements made in the “Population Bomb”, this prediction has not materialized. There is no evidence that we are going to starve ourselves into extinction any time soon. Of course, there are regions of the world, like Sub-Saharan Africa, where food insecurity not only exists but has a major impact on childhood and infant mortality and is a powerful factor in female decision making around fertility (43, 44). However, food insecurity cannot explain the world-wide fall in TFR since the mid 1960s. In fact, our global capacity for food production has massively increased as fertility rates have fallen, not the other way round (7). Moreover, some of the most dramatic declines in TFR over the past half-century have been seen in extremely prosperous nations such as Taiwan, China, South Korea, and Japan, where food has been in abundant supply.

## 5 Socio-educational factors

Whatever forces are counteracting human fertility, they are powerfully linked to global prosperity and the socioeconomic changes that accompany the generation of advanced industrial economies. Increased socioeconomic development has long been known to drive a demographic transition characterized by reductions in infant mortality and, subsequently, TFR. Of course, the world is composed of many different societies and cultures each of which will generate its own unique response to prosperity. This makes generalizing the impact of social factors on population growth extremely complex. However, we should also recognise that we are witnessing a *global* decline in fertility rates. All countries seem to be affected by this phenomenon, regardless of their individual culture, creed, or developmental status. Thus, while cultural differences are clearly important in nuancing changes in TFR at a national level, there are other powerful factors operating on fertility that cut across cultural boundaries and are inducing a world-wide change in our capacity, or willingness, to reproduce.

### 5.1 Delayed childbearing

One of the hallmarks of an advanced civilised society committed to gender equality, is the education of women and their entry into the modern workplace (16, 45). Increasingly women are working long hours away from home and, in the most advanced economies, entering occupations such as medicine, law or engineering that require years of training before they achieve the professional qualifications needed to enter the work force (46–48). A major consequence of this trend is that women are now leaving it until their early 30s to initiate a family in most modern post-transition societies (49). This delay in establishing a family, enhances the educational attainments of women and advances their employment prospects in the workplace (50). However, it also creates a significant problem because it conflicts with our basic biology (7, 51, 52).

We are an unusual species in that our capacity to reproduce is lost in mid-life due to a precipitous decline of female fertility (53). This decline is associated with a loss of developmental potential on the part of the oocyte that occurs between the ages of 35 and 42. As a result, the delay in starting a family seriously restricts the amount of time available to have any children at all. This contrasts dramatically with our hunter-gatherer forebearers who would have had their first child when they were around 18–19 years of age, when women are at their most fertile (54, 55).

Another social element in this infertility equation is that educated women are finding it increasing hard to select a mate who meets their expectations. Educated women seek partners who are at least as professionally and educationally successful as they are. Since the ratio of women to men in tertiary education is becoming more positive, there is a growing paucity of males amongst the educated members of society that might be suitable for establishing a long-term relationship and starting a family. This, in turn, drives women towards more demanding careers that reinforces their reluctance to start a family (56, 57). The increasing tendency for the female partner to be more highly educated than the male is known as hypogamy. Not surprisingly, such hypogamous couples have been found to exhibit lower fertility than their homogamous (both partners equally educated) or their hypergamous (male partner more highly educated) counterparts, although this effect is more apparent on some countries (Hong Kong, Bulgaria, Austria, Belgium) than others (France) (58–60).

### 5.2 Ideal family size

Declining fertility rates may also be influenced by changing concepts relating to the “ideal family size” which is often predicated on an individual's lived experience (61–63). This relationship between family of origin and ideal family size is widespread across different countries, particularly in those undergoing the demographic transition (64–68). As fertility rates come down across the world, so our concept of the “optimal family size” also becomes revised in a downwards direction. In China, the one-child-family policy succeeded in creating a generation of young adults for whom a single child is not only their lived experience, but their ideal (69). So, even when the Chinese Government tried to rekindle interest in procreation by introducing the two-child-family policy in 2016, and the three-child-family policy in 2021, the impact was negligible and fertility rates have continued their inexorable decline (70).

### 5.3 Developmental idealism

According to the “developmental idealism” model, as societies go through the demographic transition they are guided and motivated by the desire to create a modern society characterized by, among other things, low, controlled fertility, gender equality, mature marriage, autonomy, the independence of children and individual freedom (71–74). This model highlights a set of beliefs and values that identify the attributes of a modern society and the means of achieving these goals. It is a cultural zeitgeist that provides a trajectory for societal development and constitutes part of the complex array of social and cultural factors that contribute towards our conceptualization of an ideal family size (72, 74).

### 5.4 Additional socio-cultural determinants of fertility

Outside of family history, education and perceptions of modernity, other social factors determining fertility choices include maternal preferences for their offspring's reproductive behaviour (75, 76), the assimilative adaptation of migrants (77), the impact of religious idealism (78–80), the importance of traditional cultural values and beliefs (81, 82), the incidence of marriage (83), the changing pattern of divorce (84), ethnicity (85), and exposure to mass media (86). In addition, there has been a major cultural shift in our attitude towards childlessness, which is currently experienced by approximately 1 in 4 of women in modern industrialized societies but could dramatically increase in the future (87–89). Childlessness may also be more acceptable in societies that are no longer driven by religious groups with a strong commitment to procreation and the growth of their particular brand of evangelical fervour. For some people, childlessness is a conscious ethical decision reflecting a lack of confidence in the future and the kind of world their children would be born into. A decision to remain childless may also reflect a desire to maximize the time and resources available to become fulfilled from an entirely personal perspective. We are seeing the progressive emergence of an entire generation of young adults for whom neither procreation nor religious devotion constitutes the meaning of existence. Rather, the purpose of life is a voyage towards the attainment of self-fulfilment and the realization of potential (90, 91). This focus reflects the individualism that pervades Western culture and encourages members of society to look at life from a very personal standpoint. This ethos is not all bad. Many young people see their individual contribution to society in terms of making the world a better place for future generations and there are many examples of strong, young individuals who have the courage, motivation, confidence, and energy to stand above the crowd and make their own indelible mark on the progress of humanity.

Clearly, a complex array of social factors is involved in defining the reproductive choices made by any given couple depending on their age, wealth, nationality, cultural heritage, ambition, religious persuasion, gender, family history, mate availability and political proclivity. Whatever the reason, it should be recognised that low fertility to the point of childlessness is now the preferred lifestyle choice for many.

## 6 Urbanization and infertility

In addition to this complex array of social and cultural factors impacting fertility rates, the progressive urbanization of our species is also influencing our desire to have children because of the opportunities such environments provide for the professional advancement of women, the ready availability of contraception, myriad social distractions, the lack of physical space to raise a family and the economic cost of child-rearing (92). Urban environments are also more likely to be contaminated with reproductive toxicants, particularly air pollutants that, as we shall see later, have the capacity to suppress fertility (93, 94).

Rampant urbanization (95), changing attitudes to marriage in many countries (96), the rapid growth of single parent families (97), the rise of secularism (98), and the increased acceptance of sexual diversity in all its various forms (99), are all telling us that we live at a time of unprecedented socio-religious change. In an overcrowded, secular, urbanized, unsafe world, low fertility is becoming the socialized norm, and many will choose to remain childless as a matter of principle. Equally others will decide to have a family and should be facilitated to do so, because at a time of falling fertility, the future of our species may well depend upon it. Unfortunately, the decision to have a family is not the end of the story for many couples living in the 21st century. Increasingly, couples are having to turn to assisted reproductive technology (ART) for help in this regard (100) even though the cost, perceived stigma, intrusiveness, and ineffectiveness of such treatment, particularly in older women, may serve as a further barrier to their procreative aspirations (101).

## 7 Assisted conception and the economic cost of procreation

In 2010, Robert (Bob) Edwards won the Nobel Prize in Physiology or Medicine for the development of *in vitro* fertilization (IVF) as a treatment for human infertility (102). The enormity of this discovery cannot be understated. It has brought joy to millions of parents around the world who would have otherwise struggled to have a family and, in the process, created an industry reported to reach a market value of ∼$41 billion by 2027 with a compound annual growth rate of around 10% (103).

The cost of assisted conception therapy varies considerably between countries, depending on the extent to which the industry has been commercialized and the degree to which it is supported by government subsidies. In some countries, like France, Denmark, Spain and Israel, the level of support tends to be very good, while in others, like Albania and the USA, it tends to be bad to non-existent. According to the NCSL (National Conference of State Legislatures), the average IVF cycle in the US can cost anywhere from $12,000 to $17,000 (not including medication) while the average cost per delivery can vary from $40,500 to $77,700 depending on whether a single or double embryo transfer is undertaken (104). Since multiple cycles might be needed to conceive, it is not uncommon to encounter couples who have spent $100,000 or more on their journey through sub-fertility land. For young women thinking of freezing their eggs as a buffer against age dependent infertility, $30,000–$40,000 will be spent on treatment and storage in the USA. Clearly not for the faint hearted and a major impediment for those seeking help from the ART industry to have a family.

Critically, for women in the vulnerable 35–42 age group when natural fertility is in freefall, IVF can provide little comfort. Live birth rates following assisted conception follow exactly the same decline with age as we see with natural conceptions (105–107). This is because, all assisted conception can ever achieve is to enhance the chances of fertilization by placing sperm and egg in intimate proximity. Unfortunately, the issue for ageing women is not failed fertilization but a loss of developmental potential on the part of their eggs as a consequence of ∼40 years exposure to environmental and lifestyle factors that compromise the capacity of this single cell to be fertilized and develop into a new individual. This functional decline in the oocyte's developmental potential involves a variety of mechanisms including oxidative stress and aneuploidy that cannot be reversed by ART—even though many couples mistakenly believe it can (108–112).

Naturally, the assisted conception industry is doing its best to address this issue from a technical standpoint, including the introduction of gamete donation programs, improved oocyte freezing protocols (113) and the monitoring of embryos to ensure that they are euploid (114). However, the scale of the problem is so overwhelming that such approaches will never solve the fundamental issue: our abject failure to understand that we cannot change our basic biology. The female reproductive cycle cannot suddenly be reprogramed to accommodate our 21st century lifestyle aspirations. Rather, society has to change to accommodate our biology and provide couples with all the support they need to have their children earlier, without sacrificing their professional/lifestyle ambitions.

## 8 Age, low fertility, and compensatory immigration

Another facet of the global decline in fertility rate is that this change is occurring at a time when the miracles of modern medicine are helping us to live longer lives (115). The major consequence of this trend is that economic growth will stall and the downward pressure on fertility will increase. We cannot grow the economy, if a majority of the population are aged and are having to be supported by the labours of a shrinking cohort of young workers. Just consider the imminent ageing tsunami in China to see how difficult it will be to manage this situation in the near future (116, 117). To sustain the population, young adults are going to have to work very hard and be prepared to see a majority of their income disappear in the high levels of taxation needed to support the aged-care facilities that modern society demands. As a result, we might expect to see a perpetuation of the downward economic-demographic spiral, diminishing the financial confidence of the young, reducing their interest in, or capacity for, having a family, and further suppressing our capacity for economic growth.

On the positive side, one of the most powerful socio-political weapons we possess to stem the tide of infertility and population decline, is to promote immigration (118). This policy has been very effectively used by the major economies of USA, Australia, and the UK where 14% - 30% of the population are immigrants from overseas. These immigrants not only help bolster population numbers but also make a significant contribution to the age structure and productivity of the population, providing the immigrants are young, suitably skilled, and well-integrated into the host community. Countries like Australia and the USA are founded on the productivity of their immigrant work forces. However, in the long-term, this approach may not be sustainable for two major reasons. First, there is a strong tendency for the offspring of immigrants from high fertility countries to become assimilated and adopt the reproductive behaviour of the host nation in a form of intergenerational adaption, so that the boost to national fertility will only ever be temporary (119). Secondly, fertility decline is a global phenomenon that is impacting the “sender” nations. Even in Sub-Saharan Africa where the demographic transition is at an early stage, the trend in TFR is still, as we have seen, sharply downwards (Figures 2A–C).

In addition to the falling TFR in Africa, the Chinese population is already in decline and the Indian population is just a couple of decades behind. Clearly, the period over which prosperous nations such as the USA, Australia, and the UK, will be able to paper over the cracks of their own infertility by offering visas to young skilled immigrants from other countries has limitations. Moreover, the use of immigration to assuage the demographic demons is subject to unexpected interference from major shifts in the geopolitical landscape, unanticipated wars, and unwelcome global pandemics like COVID19. It may offer a short-term solution but, ultimately, the fundamental forces driving global fertility decline are going to have to be confronted.

The combination of social, educational, and economic factors cited above is evidently putting downward pressure on global fertility, leading to exactly the kind of low fertility trap as originally hypothesized by Lutz et al. (4). However, if population decline was simply the product of such socio-economic factors, then surely it is a problem that can be fixed, providing that governments adjust their national policy settings appropriately and their citizens are interested in procreation. Unfortunately, the major reason why population decline will be much more difficult to reverse than to induce (indeed, the reason it may be a permanent infertility trap, rather than a temporary state-of-affairs) has as much to do with our deteriorating environment as it does our socioeconomic status.

## 9 Environmental factors and infertility

World-wide, we are witnessing dramatic changes to human health affecting a wide range of conditions including breast cancer, cardiovascular disease, obesity, diabetes, autism, asthma and infertility, that are increasing at an extremely fast rate and are heavily impacted by environmental and lifestyle factors (120). The causative factors include diet, radiofrequency electromagnetic radiation, climate change, and myriad forms of chemical pollution. In rural areas, pesticides and herbicides pose a particular threat, while in densely populated urban areas, atmospheric pollution, contaminated water supplies and various lifestyle factors associated with modern society, including smoking, sexually transmitted disease, sedentary occupations, drug abuse and obesity, are impacting reproductive health (121–124). Micro- and nanoplastics are particularly concerning from a public health perspective given their recent emergence as a positive risk factor for cardiovascular disease (125) and their evident ability to impact the reproductive system (126, 127). Not surprisingly, both male and female reproductive health are vulnerable to onslaughts engineered by lifestyle or a polluted environment (125–130). The male has just received more attention than the female in this respect, because spermatozoa are more accessible to analysis than oocytes. Where such studies have been conducted, they have clearly demonstrated that male reproduction is highly susceptible to environmental change, as indicated by data revealing a recent global decline in sperm production and a parallel increase in the incidence of testicular cancer (131).

### 9.1 Falling sperm counts

All nations, East and West, seem to have experienced a decline in sperm counts as they became more affluent (132–135). This decline has been seen in China, Africa and South America as well as advanced Westernized economies (North America, Europe, Australia, and New Zealand) (134, 136–145). The decline itself is not particularly worrying because even in the most seriously affected nations, sperm concentrations are still within the normal range and there are no data to demonstrate a direct impact of declining sperm numbers on our capacity to reproduce since the average human ejaculate still contains around 50 million spermatozoa /mL (146). Moreover, sperm counts are a very unstable basis on which to project any kind of secular trend. They are powerfully influenced by inter-ejaculate variation due to factors such as ejaculation frequency, psychological stress, sleep deprivation, obesity, viral infections, and lifestyle factors such as tobacco, alcohol, and drug abuse (147–150). Furthermore, the data supporting the falling sperm count hypothesis have been cross sectional in nature and therefore prone to additional variation due to differences in collection methods, laboratory protocols, replicate number, geographical location, and patient selection criteria as well as the structure, size, and ethnicity of the sperm donor populations (151–153). Given all these potential sources of heterogeneity, it is not surprising that some conflicting data have been generated that have led to doubts over the veracity of the falling sperm count hypothesis (154, 155). Nevertheless, we would expect the imperfections inherent in such datasets to obfuscate any secular trends relating sperm counts to time, not create them. The fact that such trends have been observed in a majority of published data sets as well as the strength of recent data (135) suggests that the decline in sperm counts is a real phenomenon that appears to be accelerating with the passage of time. Linear extrapolations of such data to suggest that half the men in advanced modern economies will have close to no sperm at all by 2045, are naturally fraught with uncertainty (156, 157). However, unless we manage to identify the underlying causes of this phenomenon, we have no reason to doubt that sperm counts will continue to decline to the point that fertility is compromised (157). Determining the cause of this change in semen quality is therefore paramount.

A plausible hypothesis, which is the focus of much ongoing research, is that sperm counts are declining as a result of environmental contamination with foreign industrial chemicals and agricultural pollutants (xenobiotics) that have estrogen-like activities; the so-called environmental xenoestrogens, such as phthalate esters and bisphenol A (158). In animal models, the impact of these chemicals on reproductive function is incontrovertible (159, 160). In a human context, the threat might come from a combination of such industrial pollutants, mixed with natural or synthetic estrogens present in our food and water or generated endogenously as a consequence of factors such as obesity. One of the consequences of such a combined estrogenic attack might be a lowering of testosterone levels (161–163) and, in this context, it may be significant that age-adjusted circulating testosterone levels also appear to be reducing with the passage of time in concert with the progressive decline in sperm counts (7, 164–166). In addition to testosterone decline, we should also recognize that several pollutants, including members of the bisphenol family, have anti-androgenic effects that further erode the ability of testosterone to support reproductive function (167). A causal association between raised estrogen levels, declining testosterone and reduced sperm counts is certainly feasible, and could involve a variety of developmental and metabolic pathways (168). If this is the case, and environmental, synthetic, and natural estrogens are conspiring together to reduce testosterone levels and thereby impair sperm production, we can expect the latter to continue to decline in the future. Unfortunately, controlling levels of estrogen exposure is not a major part of any government's environmental or health policy agenda. So, the secular decline in sperm counts, if it continues unabated, may well make its own indelible contribution to the infertility trap enveloping our species.

### 9.2 Testicular cancer

The global decline in sperm counts is not the only evidence that environmental factors, particularly xenoestrogens, are having a major impact on male reproductive function—consider what is happening to testicular cancer (7, 169). Throughout the developed world, testicular cancer rates have been increasing at unprecedented rates. It is not, like prostate cancer, a function of the increased longevity that characterises advanced industrialized nations. It is also not a cancer that has miraculously appeared because we now have better methods of diagnosis. It is a cancer of young men exhibiting a peak incidence around 30–34 years of age and associated with readily detectable changes in testicular size or shape and, occasionally, the onset of pain in the testes or lower abdomen.

The UK has seen a 27% increase in testicular cancer incidence rates since the early 1990s and is currently witnessing the detection of 6 new cases a day (170). In other advanced economies such as Australia, testicular cancer is now the most common cancer in young men and the trajectory is strongly upwards (171). Similarly in the USA, testicular cancer rates have risen to become one of the most common cancers in young men and the trajectory is in the ascendency, with an anticipated 9,190 new cases in 2023 (172). Moreover, across the globe, testicular cancer rates are highly correlated with national prosperity as reflected in per capita GDP (7, 8, 173, 174).

So, the wealthier a country becomes, the higher the risk of contracting testicular cancer. This particular cancer is therefore mirroring the impact of affluence on fertility rates and sperm counts. A plot of TFR against testicular cancer rates demonstrates that as the former fall towards replacement levels, the incidence of testicular cancer rises dramatically (7, 8). This does not mean that the global decline in TFRs is directly related to the increased incidence of testicular cancer. The latter is a mercifully rare condition that, even in the most vulnerable of countries, has an incidence of only around 1 in 10,000—too uncommon to create any demographic damage directly. However, an increasing incidence of testicular cancer, falling TFR and declining sperm counts are all independent attributes of socioeconomically advanced societies reflecting the changes in lifestyle and environment brought on by affluence in association with the demographic transition. Moreover, both the decline in sperm counts and the increase in testicular cancer (and plausibly, the global increases in breast and uterine cancer as well) seem to be dependent on the presence of environmental estrogens (175, 176). There are even data to suggest that such compounds are responsible for distorting the sex ratio at birth, leading to a relative reduction in the number of male offspring (176). Worryingly, these trends seem to be progressing relentlessly and show no sign of stabilization.

## 10 Genetic determinants of human infertility

Another potential consequence of the demographic transition is an impact on the overall fecundity of our species. This topic lies at the interface between biology and demography and has been difficult to disentangle for a variety of reasons (177). In order to understand this complex issue, it is first important to emphasize the distinction between fecundity and fertility rate. In biology, the fecundity of an individual represents the probability of conception in a given menstrual/estrus cycle. It is a basic biological concept that is powerfully impacted by genetic and environmental factors and defines our fundamental capacity to reproduce (178). Fertility, on the other hand, is an output measure; it refers to the number of offspring that will be born during a woman's reproductive lifespan and is influenced by contraception and a complex array social, educational, economic, and cultural influences discussed above, none of which are not directly heritable.

In feral animals, reproductive fitness is maximized to deliver the largest possible number of young in a reproductive lifetime. If we take deer as an example of such a feral species, fecundity is generally extremely high, typically over 80% (179–182). Under these circumstances any genetic or epigenetic change that compromised fecundity would be rapidly selected against and deleted from the population. Our species is different. We generate neotenous, immature young that require many years of postnatal care to reach maturity. As a consequence, there has been a quality-quantity trade-off during human evolution, with the result that long-term reproductive fitness requires only a moderate level of fecundity (183), calculated to be around 20%–30% per cycle (184–186). This fecundity setting may be relatively low but has been sufficient to allow women during the earliest stages of human evolution to produce 5 or more children at a time when life was nasty, brutish, and short (186, 187). Since at least half of these children would never reach sexual maturity and have children of their own (188), birth and death rates more or less balanced and allowed human population numbers to remain relatively stable throughout much of history.

The high incidence of infant and childhood mortality during the early stages of human evolution ensured there was constant selection pressure on keeping fecundity at this optimal level. A couple could only pass their genotypes onto the next generation if they, themselves, were capable of having 5 or more children. If they were only able to have 1–2 offspring, their lineage would not have lasted long. Fast forward a few millennia and you have our current situation where, in affluent society, high fertility is neither sought nor required. As a result of the demographic transition, we are now no longer selecting for high fecundity genotypes. There is no point. We no longer need children in our workforce, and we no longer have to cope with high rates of infant and childhood mortality. Furthermore, the widespread use of contraceptives coupled with a general lack of procreative desire in post-transition societies, effectively neutralizes any selective advantage high fertility genes might have had (189). Given this lack of selection pressure on reproductive capacity in modern society, are we losing high fertility genotypes and becoming less fecund as a consequence? The answer depends on when during the demographic transition you look.

One of the features of the demographic transition is that in advanced economies, the fertility change has been extremely fast. If, for example, we look at the tiger economies of SE Asia (Hong Kong, South Korea, Taiwan, Singapore), it took an average of just 20.5 ± 2.02 years (barely one generation) to go from a pretransition TFR of 5.672 ± 0.167 to sub-replacement levels (>2.0). In China the equivalent journey took 23 years. Even Albania, with no help from contraception at all, managed the feat in 42 years. Of course, the descent to sub-replacement levels of fertility may take longer in some countries than others. However, these data indicate that, under appropriate conditions, the decline in TFR can be so rapid that it cannot be driven by genetic factors: it is almost exclusively due to changes in human behaviour occasioned by differences in education, age, mate selection, marriage, developmental idealism, cost, professional ambition etc. discussed above.

However, once sub-replacement TFR levels have been achieved, we enter into a post-transition phase of demographic development (sometimes known as the Second Demographic Transition) when genotypes compatible with a TFR above 5 are still there, but now the selection pressures that forged their existence have dissipated. In the absence of significant selection pressure in modern society, it would be logical to assume that the post-transition era will be characterized by a gradual deterioration in the genetic underpinnings of our fecundity. Such genetic changes would not only reinforce the reduction in TFR associated with the demographic transition, but may also prevent any subsequent reversal of this process, even if the socio-political will should be there to do so.

Whether, at the present time, the Second Demographic Transition has been sufficiently prolonged to observe a significant impact on human fecundity is very difficult determine because TFR is so profoundly impacted by myriad cultural social, economic, and political overlays (177, 190–192). A progressive increase in the time taken to achieve a pregnancy (193) in association with the increasing demand for infertility treatment amongst young adults as well as declining semen quality, are all suggestive of a decline in fecundity, however these trends do not amount to definitive evidence (133, 191). Indeed, superficially, it might be argued that selection will always favour those phenotypes with propensities towards higher fertility, and thus fecundity should not be impacted following the demographic transition. Some authors have even argued that the relaxation of selection pressure associated with the demographic transition may actually lead to an increase in fertility because people with high fecundity genotypes will be making a disproportionate contribution to the next generation (194). The fundamental problem with this argument is that post-transition, TFR is determined by a range of contextual factors that are not directly related to our fundamental fecundity (6). In this situation, parents with high fertility genotypes may still decide to limit themselves to one child for all the socioeconomic/cultural reasons highlighted above, or couples with low fertility genotypes may decide to have three children with the help of ART. Thus, following the Second Demographic Transition, the upper limits of even a high-fertility-genotype couple's fecundity will not be tested and will confer no selective advantage. The inevitable result of such a relaxation in selection pressure will be the progressive accumulation of poor fertility genotypes.

Of course, natural selection will always weed out mutations that cause a serious loss of fertility or sterility; the more severe the phenotype, the stronger the negative selection. This is undeniably the case with the three major genetic causes of human infertility: microdeletions on the Y chromosome, Klinefelter syndrome (XXY) and Turners syndrome (XO) (195, 196). These genetic conditions arise spontaneously in the parental germ line and because the phenotype is so severe (they generally induce complete sterility) they are heavily selected against because their appearance is literally the end of the line. The fact that these conditions collectively comprise the major genetic causes of human infertility, suggests that at the present time there is significant genetic upheaval in the germ line. To counter such turmoil, natural selection is still operating to reduce the incidence of mutations that dramatically impact human fertility (197). However, the selection pressure on mutations that induce *subfertility* (rather than completely annihilate reproductive fitness) is not as intense in post-transition societies. On the basis of these considerations, it is hypothesized that the relaxation of selection pressure on such suboptimal fertility genotypes is allowing the accumulation of myriad genetic defects involved in the aetiology of human infertility. In support of this hypothesis, there is already emerging evidence for an increase in genetic variance in populations that are experiencing sub-replacement levels of fertility (198).

Further to such genetic considerations, we should not forget the potential impact of epigenetic changes on fertility. A variety of lifestyle and environmental factors are known to impact the epigenetic status of the germ line including obesity (199) and environmental toxicants (200). Via such means, the germ line is able to respond dynamically, and sometimes aberrantly, to changes in the parental environment by inducing alterations in patterns of DNA methylation and small non-coding RNA species within the male and female germ lines (201, 202). Whether such changes are limited to one or two generations or will have a long-lasting impact on human fecundity is, as yet, unknown.

## 11 Sperm DNA damage, IVF, and infertility

In this dynamic situation, the fires of genetic drift are being fuelled by environmental and lifestyle factors that are inducing high levels of DNA damage, particularly oxidative DNA damage, in human spermatozoa (203–206). The appearance of such genetic damage in spermatozoa is hypothesized to result in an increased mutational load in the offspring, at least 75% of which is known to be paternal in origin (207). Overall, the active generation of *de novo* mutations via the male germ line, coupled with the relaxed selection pressure typical of modern industrialized societies, is a perfect storm that is already impacting human fertility (207, 208) and may have significant implications for our reproductive fitness on the road ahead. In this context, the fact that we are reproducing at later ages, does not help. As men age, they accumulate progressively more DNA damage in their spermatozoa (202) while the oocytes of their ageing partners are gradually losing their capacity for DNA repair (209). The result of this combination of factors is an age-dependent increase in offspring mutations, some of which may subvert fertility (207).

Paradoxically, the relatively high incidence of human infertility may also be accelerated by the indiscriminate use of assisted conception therapy. With the passage of time, IVF and particularly its highly invasive variant, Intracytoplasmic Sperm Injection (ICSI), have become default treatments for many different types of infertility. In some countries around 10% of the population are now generated using such procedures and in all countries the vector is steeply upwards (7). Logically, the large-scale uptake of ART will, by allowing poor fertility genes to be retained within the population, further compromise the fecundity of our species (207, 210). Naturally, at an individual level it is important that couples are not discouraged from seeking assisted conception therapy if their clinical situation warrants such treatment: maintaining a humanistic individualism perspective is critical in such circumstances. However, from a species perspective, the indiscriminate large-scale use of ART may ultimately create more problems than it solves.

In addition to the cost of meeting the projected future demand for assisted conception services, if the State leaves such provision to the private sector, then major ethical issues will be raised concerning the ability of financially disadvantaged citizens to gain access to such treatment in the future. We cannot live in societies where only the rich can procreate. The application of ART at scale will also have implications for national health services, above and beyond the rising tide of infertility. There are several congenital, pathological conditions created by this form of therapy (211), the resolution of which will make further demands on the health service. In addition, the commercialization of ART has meant that questions are no longer being asked about the cause of the infertility and thus whether less invasive techniques, that do not place the entire burden of treatment on the female partner, might be developed to solve a couple's infertility problem. We are still in the early stages of the ART revolution and have not yet reached any kind of crisis point. However, if we do not recognize these trends, and allow the status quo to persist into the coming decades, then this moment will arrive. Ultimately, health service providers should be aware that the more we use assisted conception in one generation, the more we are going to need it in the next.

## 12 Summary, conclusions and future prospects

So, as societies become more prosperous, there are impacts on fertility from all sides:- social, economic, environmental, and biological. Skilled labour shortages and crippled aged care sectors are just the beginning. Managing the social, political, and economic ramifications of uncontrolled population decline as well as the concomitant shift in age structure will be extremely challenging. So, what can be done?

From a socioeconomic perspective, encouraging more women into paid employment is not just an ethical imperative, it is an economic necessity. Such a strategy would make a massive contribution towards generating the kind of productivity needed to support the inverted population age pyramid typical of modern industrialized societies. The problem is that the more women are engaged in driving the economy, the less likely they are to have children, thereby reinforcing the downward fertility spiral. Finding ways of supporting young couples to have children and simultaneously contribute to the economy is the dilemma of the age. Raising the retirement age (212, 213) and encouraging more disabled people into the workforce (214) should help spread the workload. The widespread implementation of Scandinavian-style parental support schemes, baby bonuses, income tax reform, the increased provision of affordable housing, and a greater emphasis on regional development, should all be part of finding a solution to this emerging problem (215–217).

However, even if we can successfully address such socioeconomic issues in the short-term, there are still long-term factors in the form reproductive pollutants and the loss of high fertility genotypes from the population that will continue to challenge us in the post-transition era. Such trends are sweeping the world in the wake of increased global prosperity and placing downward pressure on human fertility that might become difficult to reverse. Genotypes cannot be suddenly changed; genetic constitutions coding for optimal levels of fertility evolved over thousands of years and allowed us to survive in the earth's harshest climates. Once those genotypes are diluted out, we shall not have the luxury of millennia to engineer their return and shall have to face the re-emergence of harsh environments in the wake of accelerating climate change, without the support that a highly evolved genotype brings.

To counteract such long-term changes, it is critical that we prioritise and resource reproductive health as a discipline. Although infertility is not a life-threatening condition, improving our understanding of this condition to the point that individuals are provided with the means of exercising their reproductive choices, whatever they may be, is an important aspiration for the field. Environmental Protection Agencies, the world over, need to be much more vigilant about identifying and removing reproductive toxicants from our environment. We also need to understand much more about the fundamental etiology of human infertility and stop using assisted conception as a default treatment for every clinical case that we confront. In addition, the general populace should be more aware that their fertility is not something that can be taken for granted. Reproductive health is fragile; it will respond to a healthy lifestyle but can be ravaged by many factors including time, diet, drugs and the environment. It is certainly within our power to solve the fertility problem and build a world characterized by sustainability, inclusivity, and equality, where personal fulfilment can be attained without threatening the future of our species, or our planet. If we can manage the process, an orderly transition to a lower global population size may bring many social, environmental, economic, and clinical advantages (218). However, the ubiquitous occurrence of the demographic transition and the self-propagating, unrelenting nature of the social, environmental, and genetic factors suppressing human fertility in modern society will pose a challenge that can only be met by increased awareness of the change that is about to come.
